# The Physiology and Pathology of the Second Dentition

**Published:** 1891-08

**Authors:** Richard Cole Newton

**Affiliations:** Montclair, N. J.


AMERICAN JOURNAL

—OF—

DENTAL SCIENCE.

Vol. XXV. Baltimore, August, 1891. No. 4.

ARTICLE I.

### THE PHYSIOLOGY AND PATHOLOGY OF THE SECOND DENTITION.

BY RICHARD COLE NEWTON, M. D., MONTCLAIR, N. J.

(Read before the New Jersey State Dental Society, at its Annual Meeting, 1890.)

A year ago I had the honor of addressing this learned Society on the subject of "The Teeth as a factor in Diagnosis." Naturally, as a clinician, I looked at my subject from a practicing physician's stand-point. The discussion, however, which my paper excited, showed that the dental profession is fully alive to the economic importance of the teeth, and has noted and studied the peculiarities in these organs which are caused by different cachexiæ and various morbid states of the system, whether hereditary or acquired. Having been doubly honored by a second invitation to read a short essay before you this year, it has occurred to me that one might with profit take up another phase of our subject of last year and consider the physiology and pathology of the second dentition somewhat in detail. When we reflect that the infant comes into the world with the rudiments of four permanent teeth, we may fairly say that the physiology of the second dentition begins in intra-uterine life and terminates with the eruption of the wisdon teeth, say at twenty years of age; we have then to do with the most important third of human life, at least from a physiological point of view, and a thorough discussion of our subject would take us over a great extent of ground, much of which is already familiar to you. I will not, therefore, weary you with an elaborate discussion of the physiology of the first twenty years of life, but will endeavor to point out the relation that the process of the second dentition bears to the general health, and will supplement this with a few remarks on the hygiene of this period, which I trust will not be without interest.

Human life has roughly been divided into periods of seven years, and it is estimated that the whole body will be practically transformed by the slow process of elimination and substitution in about that period of time. The period of life from birth until the completion of the second dentition naturally divides itself into three periods of about seven years,—viz., first, until the complete eruption of the first four molars of the permanent set; second, until the complete eruption of the second four molars of the permanent set; and third, until the complete eruption of the third four molars. Of course these periods are subject to considerable variation, especially the last; but they are constant enough for the purpose of discussion, and afford convenient divisions of our subject. The first period, *i. e.*, from birth to seven years of age, is, I am inclined to assert, without fear of contradiction, from a physiological stand-point, the most important period of human life, and I believe that it can be shown that the care of the child up to seven years of age has more to do with his subsequent development than any other factor, heredity, perhaps, excepted. During this period the crowns of all the permanent teeth, except the third molars, are formed. The brain experiences over two-thirds of its complete development. The child has mastered the use of his senses, has learned to reason, has learned an elaborate and difficult language; and has often learned to read, etc. In short, in no other seven years of our earthly existence is a corresponding advance made. Now, what marks the conclusion of this period? I reply, the complete eruption of the first molar teeth of the permanent set. Owing to the varying lengths of time that different children take to reach the same stage of development, the eruption of these teeth affords a far better and safer criterion of the completion of the first physiological period of life than the mere lapse of months or years. By the English factory laws a license to work full time is not given to the youth of either sex until the second four permanent molars are completely through. This has proved to be a more reliable test of physical capacity and endurance than age or statue, since often the tallest and largest children are really the most immature and frail. Nor is it unnatural that it should be so. *Ceteris paribus*, it must take longer to develop a large body than a small one, and the old fashioned phrase, "outgrown the strength," like most popular notions, has a foundation in fact. Naturally the development of the first four molars has less interest from a commercial point of view, but it undoubtedly marks as important a stage in bodily development as does the eruption of the second molars.

We see, then, that the complete eruption and occlusion of the first molar teeth mark a very important era in human life. The brain has attained very nearly its growth, although it increases slowly up to twenty or even thirty years of age. Its growth subsequent to seven years of age is much slower than previous to that time.

The body has also at seven years of age attained about two-thirds of its height and over one-third of its average weight, if its processes have gone on normally and I think I have said enough to show that the complete eruption of well-formed first molars is the most important criterion and sign of a normal development of these great powers and a completion of the first great division of human life.

The second physiological period is, like the first, ended by the complete eruption of four molar teeth,—viz., the second permanent molars. During this period the rudiments of education are secured, the character is for the most part formed, etc. By fourteen years of age a pretty fair estimate can be obtained of the way in which the child will turn out physically and mentally. Are there any especial features that interest us as clinicians in the second dentition, especially from the seventh to the fourteenth year?

The late Dr. James Jackson, of Boston, in his well-known "Letters to a Young Physician," dwells at some length upon the diseases of the second dentition, as does also in another place our distinguished countryman, Dr. George B. Wood. As there are those who deride the considerable importance accorded to the first dentition from a clinical stand point, no doubt there will be very many who will assert that there is little or nothing to be said in the matter of the clinical importance of the second dentition, but I think it can be easily proved that the latter class seriously under-estimate the significance of the second as well as of the first dentition.

Mr. Jonathan Hutchinson says that the first four molar teeth are the test-teeth of a condition in children which leads to the formation of cataract, and which is also characterized by weak teeth, deficient in enamel. He says that there is no especial alteration in the form of the teeth, nor is the enamel uniformly defective and soft. It is wanting in irregular patches. There is perhaps no man living who has more carefully studied these questions on diathesis, development, and heredity than this distinguished Englishman, and the great value that he has been led to attribute to the teeth as marks and signs of constitutional vigor, or its opposite, and of normal, abnormal, or retarded development, is, to my mind at least, a marked tribute to the great importance of the line of inquiry upon which we are now engaged, and which, as I said before, has not as yet been sufficiently followed up.

Of course, all divisions of human life into periods of years are merely arbitrary. A man's earthly existence is not like a ball player running his bases. We do not reach one period of life, then stop running and wait for another chance. We advance pretty steadily from the cradle to the grave, developing up to our prime, and then gradually deteriorating until our enfeebled bodies will no longer sustain life. Of course some advance by more rapid strides than others, just as one man may run faster than another. Some children at five are as well developed as others at seven, and I believe that it is by the eruption of the first permanent molars that we are to judge of the completion of what we may call the first great physiological period of life. If all goes normally and well, "the six-year-old molars" should appear at six years, should pierce the gums without too great constitutional disturbance, should not be soft and carious, but firm and well formed, and the child should almost be unaware of their arrival. If all this happens, we may assure the parents that the child promises to grow up healthy, and may be now sent to school. Dr. Anstie, Dr. Day, and others have pointed out the evils of sending children to school too early and putting upon the system, already taxed nearly to its utmost, the additional strain of study, confinement, and the bad air of the schoolroom. No young child should be urged or driven to study, and precocious children under nine or ten years of age should be held back rather than pushed forward.

If, however, the teeth are not normally erupted as to time, and are soft and perishable; if their eruption be attended with convulsions or other constitutional disturbance, the sign is as plain as a pike-staff that the child should be immediately put under a doctor's care. It is absurd and even criminal to attribute to the visitations of Providence what we have brought upon ourselves by our ignorance and folly. I ventured to assert above that the proper care of children during the first seven years of life has more to do with their subsequent health and development than any other factor, hereditary alone excepted; and this, I believe, can be proved. It has not been proved upon a large scale, because it is not as yet possible to bring up large numbers of children physiologically. When, if ever, the flattering dreams of Mr. Edward Bellamy are realized, and individual life is merged in that of the community, perhaps children can be properly brought up. At that time the doctors will perforce become communists,—because there will be so little for them to do. Nature has given us in children's teeth a sign which we cannot afford to neglect.

I think it can be easily shown that many of the morbid conditions and diseases of adolescence are due to the second dentition. In addition to the distinguished American physicians whose names I have just mentioned, I can give a number of others who attribute serious and even fatal maladies to the second dentition. J. Russell Reynolds says that there is no doubt that epilepsy may be caused by the second dentition. Erb and Heine mention teething as a cause of poliomyelitis anterior and spinal meningitis, and many other writers speak in a similar strain. Dr. Louis Starr has recently published a paper in the *Therapeutic Gazette* on the diseases of the second dentition and their treatment. Several writers, among others Dr. Mulveany, of Brooklyn, have called attention to the association of hip-joint disease and the eruption of the first permanent molar teeth, the majority of cases of this complaint showing themselves between the fifth and seventh year. Dr. Mulveany asserted that he had never seen a case that did not begin during the period mentioned. He also gives some interesting cases of pseudo hip-disease in small children, all of them evidently too young to simulate the complaint. In one of these, so good a surgeon as Dr. Sayre is said to have been so completely deceived by the symptoms and history of the case as to have advised an exsection of the head of the femur and actually to have cut down with the intention of removing this part of the bone, only to find that the joint was in a healthy state.

The cases all recovered readily after the complete eruption of the teeth. Dr. Starr says that the anterior molars generally give rise to more marked disturbance than do any of the teeth that replace the temporary set.

What Eustace Smith calls "mucous diseases" are more generally due to the second dentition than to any other cause or causes. I quote Professor Starr in the following description: "In this condition there is a nervous flux from the whole internal surface of the alimentary canal, which mechanically interferes with the digestion and absorption of food and greatly impedes nutrition. It may be met with at any time during the eruption of the permanent teeth. The affected child is emaciated and weak. His face is pale, though subject to marked changes in color. At times a circumscribed crimson flush appears on one or both cheeks. Again there is so much pallor, particularly about the lips, that fainting seems imminent. The eyes are surrounded by bluish circles that deepen when the face pales: the conjunctivæ are muddy and there is occasional squinting. The skin is sallow, dry and rough, and the hair lustreless and faded. There is decided pallor of the oral mucous membrane. The tongue is flabby, indented by the teeth, and glazed, and the breath is heavy and fetid. The appetite in the beginning fails, then becomes capricious, and finally is almost insatible. Eating is followed by a sensation of drowsiness and by eructations of flatus and acid liquid. Tympanitic distention of the belly and pain are always present.

"Pain may be general when it amounts to a little more than a sensation of soreness, or, more frequently, it is limited to the left hypochondrium, and then is stitch-like in character. Either variety may be constant, or present only after meals; in the former case there is temporary increase of discomfort after eating. Paroxysms of severe pain about the umbilicus, with great pallor of the face, are sometimes observed. Constipation of the bowels is usual and the evacuations which take place at intervals of two or three days are scanty and composed of small, hard, dark-colored lumps with a quantity of mucus. By day the patient suffers from headache, is languid and ill tempered. At night he is restless, grinds his teeth, starts from sleep in terror caused by frightful dreams, screams or laughs incoherently, and for a time seems unconscious of his surroundings. Somnambulism and nocturnal incontinence of urine are quite common.

"There is no alteration of the temperature, the pulse is feeble, and there is frequently a slight dry hacking cough, unquestionably due to reflex irritation of the larynx. The urine is diminished during the continuance of severe pain, but is voided in large quantities at the termination of the paroxysms.

"At intervals of two or three weeks violent vomiting and purging occur. During these attacks, which last from one to three days, a large quantity of mucus is expelled. There is slight fever, and the tongue becomes less flabby, more pointed, and covered with a thick, white, feathery fur, except along the sides, where several smooth, red, glazed patches of variable size and shape with irregular indented margins appear. The clearing-out process is followed by temporary improvement, but the symptoms slowly return and grow worse to culminate in another attack. The course of mucous diseases is very protracted, extending over months, and, while there is no regular progression, the symptoms tend to grow more and more serious as time lapses.

"Hacking cough, emaciation, and debility may suggest tuberculosis. A diagnosis, however, can be readily made by noting the state of the tongue, the mucous stools, the color and condition of the skin, the absence of pyrexia, except in the attacks of vomiting and purging, the periodicity of these attacks, the diurnal drowsiness and nocturnal terrors, and the irregularity of the course. At the same time, the outset of the symptoms after the commencement of second dentition is a point of importance.

"Mucous disease is not dangerous in itself, though by reducing the strength it predisposes to other and much more fatal diseases."

In another place Professor Starr says, "Diarrhœa is a very constant attendant upon second dentition. It is most apt to arise in the changeable weather of spring and autumn, and again the general depression produced by the coming of the second teeth would naturally favor the development of any constitutional tendency; and, having traced the connection in several instances, I have no doubt that not a few cases of tubercular ulceration of the bowels owe something to this 'etiological factor.' "

I have quoted at such length because the above is a good, even a graphic, description by an eniment clinician and writer of a common complaint of childhood which is generally referred to almost any cause but teething, as, *e. g.*, worms, the approach of puberty, malaria, etc. Dr. Jackson wrote that, since having satisfied himself of the etiological importance of the process of the second dentition, he had almost given up the use of worm-seed and other reputed vermifuges, because in his opinion most of the troubles imputed to worms were really due to something else.

A case is given of a boy of about twelve years of age who developed a chorea which lasted for several months and increased in severity; finally the lad fell into an epileptic fit. An examination of his mouth showed the gums over the second molars to be swollen and tender, and a free lancing of these finally relieved all of his symptoms. Fleiss gives a case of a small lad aged about five, who was preparing to cut his first molars; his anterior milk molars were lying half decayed in the gums, and near them were the edges of the permanent molars in a row. After a night of great feverishness, restlessness, and grinding of the teeth, the chlld arose in the morning with the left arm completely paralyzed. It was determined to extract all the carious teeth, in the hope of relieving the paralysis. However, the child was killed on the same day by an accidental fall upon his head. A post-mortem examination was held and showed, apart from the fracture of the skull, great congestions extending from the roots of the brachial nerves on the left side to the shoulder, neck, and face. "There appeared to be no doubt that dentition had produced this state of the veins." We should note in passing a curious symptom and its consequences. Irritation of the gustatory branch of the fifth nerve perverts or destroys the sense of taste. This nerve, being the one principally involved in the process of dentition, is already in a state of irritation. The loss of taste takes away or transforms the appetite. The want of food or improper food may in its turn bring on chlorosis or hysteria, and indeed any of the complaints due to improper nourishment of the nerves, a condition in young women usually ascribed to anything except the teeth.

Concerning the nervous disorders of the second dentition, at the risk of wearying you I will quote again from Professor Starr's paper. He says nervous disorders, both sensory and motor, are encountered. "Headache is common; the pain is usually temporal and unilateral. It may be scattered, however, in the occipital region or in different parts of the face, and sometimes shifts suddenly from the temporal to the occipital region. It is lancinating, more or less constant, with no distinct intermission, and during its continuance there are restlessness, anorexia, a frequent hard pulse, sweating, dilatation of the pupil on the affected side, and perhaps dimness of vision, diplopia, and colored or uncolored spectra. One or more painful points can often be detected, and generally there is a hard, tender moderately enlarged lymphatic gland in the sub-maxillary or cervical region.

"These attacks are due to disordered vaso-motor innervation, depending upon irritation of the sympathetic nerves and producing irregular contractions or spasms of the vessels (the temporal or occipital artery as the case may be). The real source of irritation is to be found in the mouth. The mode of action may be twofold. First from a swollen gum or carious tooth. The lymphatic vessels readily convey irritating matter to a neighboring lymph-gland, and the irritation here excited acts in its turn as a disturber of the sympathetic nerves, which furnish the vaso-motor supply to the carotid artery and its branches. This theory of the production of migraine has the value of the support of Lauder Brunton. The other method of production, and this I simply submit for consideration, is one of direct nervous connection, the sub-maxillary ganglion acting in the case of the lower teeth, and the spheno-palatine in the upper as the medium of transfer of irritation to the vaso-motor nerves. The motor disturbances, while not so common as the sensory, are more varied. Reflex spasm and paralysis of the eyelid have been noted. More extended paralysis also occurs. Dr. Brunton says, 'Sometimes, however, paralysis occurs of a much more extensive character, in consequence of dental irritation, especially in children. Teething is recognized by Romberg and Henoch as a frequent cause of paralysis, appearing in children without any apparent cause. According to Fleiss, paralysis of this sort occurs more commonly during the period of the second dentition, whereas convulsions generally occur during the first. Sometimes recovery is rapid, but at other times the limb atrophies and the paralysis may become associated with symptoms indicating more extensive disturbance of the spinal cord and brain, such as difficulty of breathing, asthma, palpitation, distortion of the face and squint, ending in coma and death.'"

Besides the intestinal catarrh, already noted in growing girls, catarrhs of the vaginal mucous membrane have occurred, and have led to serious charges against the purity of the patient and to much family unhappiness. Dr. Mulveany, already quoted, has given an instance in a girl of ten years, which disappeared upon *lancing the gums*, and instituting the simplest detergent treatment.

The same author gives a striking instance of enlarged cervical glands due to cutting the second molar teeth and the ease with which the true cause of such affections may be overlooked. A lad of fifteen, apparently in good physical health, was brought to a dispensary with the cervical lymphatic glands in the left side of the neck enormously swollen. The surgeon who brought him desired the opinion of his colleagues as to the advisability of dissecting out the glands. After considerable discussion the patient having been examined by the other surgeons present, Dr. Mulveany inspected the mouth carefully and found that the upper and lower second molars on the affected side had not yet protruded and were irritating the gums. The boy thereupon volunteered the information that about a year previously the glands on the other side of his neck had been similarly affected, and that he had consulted some medical man who had cured (*sic*) him.

It is hardly necessary to add that the lad escaped a difficult and dangerous operation.

Many young girls shows decided hysterical symptoms previous to the eruption of the second molars which are generally attributed to the approach of puberty. To the lay mind, at least, the approach of womanhood covers a multitude of pathological sins, and the allegation of this as cause of any symptom or set of symptoms is generally quite satisfactory. Braxton Hicks has pointed out that puberty is due to a slow development rather than a sudden change. The female genitalia begin their growth *in utero*, and it is rather that their complete development is announced by the amplitude and rounding of the figure than that the essential organs of womanhood spring into complete and perfect existence just at the time when the form is beautified and the menstrual function established. In other words arrival at puberty is only the completion of a stage of human development, just as the eruption of teeth is, and generally takes place shortly after the complete eruption of the second permanent molars. Indeed, it may be retarded or rendered painful and difficult by interference with dentition.

It is certain that cases of hysteria, chorea, and even epilepsy are cured by lancing enlarged and tumefied gums over advancing permanent teeth, and the assumption that the difficult or retarded menstruation is due to the disordered state of the system caused by dentition is more in accordance with acknowledged pathological facts than that the disordered state of the system is caused by the delayed menstruation.

Dr. Garretson remarks, in his Oral Surgery, that while dentition is a physiological process, still it is like that of utero-gestation, one of continuous irritation. While I readily admit that the disorders and disturbances of teething are not solely due to the formation and eruption of the teeth, and I believe them to be chiefly due to the perfecting of important physiological changes in the brain, stomach, glandular and osseous tissues, going on simultaneously with the eruption of the teeth, I assert, without fear of contradiction, that the normal and peaceful eruption of healthy teeth is our best visible criterion of the progress of these processes and of the healthfulness of the system, and, on the other hand, delayed and irregular eruptions of the teeth as surely evince some constitutional vice or evil hygienic condition.

Dr. Mulveany, from whom I have already largely quoted, cites a case of a young woman, fifteen years of age, who had suffered for years with a catarrhal ophthalmia which finally readily yielded to treatment after twenty-eight permanent teeth had been cut, to be followed in a fortnight by a copious, offensive purulent discharge from the vagina accompanied by excoriations from the mons veneris to the anus, which naturally led to suspicions of unchastity. However, the simplest treatment cured the condition in a few days, and the girl menstruated for the first time. Here was a case of catarrhal diathesis with local manifestations in the conjunctivæ kept up by the irritation of dentition, which persisted in spite of treatment until after the eruption of the second molara. The dyscrasia then showed itself in another region, provoked by the unusual activity of the uterus (about, one might say, to enter upon its life business); after the function of this latter organ has been established the vaginal catarrh disappeared.

It is doubtless true that many morbid symptoms really due to dentition are falsely ascribed to the approach of puberty in both sexes. It is not asserted that the former is all-important, or even more so than the latter. It is only claimed that as a factor in the production of the diseases of childhood and adolescence dentition has not received due recognition.

Any unused function is gradually lost. We use all our muscles too little; our chests are narrow and our lung expansion small because we do not exercise our arms and our lungs enough. Our teeth are poor and our jaws narrow because we do not chew enough. Ambrose Pare spoke of a "rusty filth which attacked and destroyed the teeth and was chiefly due to the omittings of their proper duty—that is, chewing."

I asserted in the early part of this paper that proper care and food in the first seven years of life would, in my opinion, have more influence upon subsequent development than any other factors whatever, heredity alone excepted. Perhaps the statement was somewhat extravagant. Let us consider it. Of course, by proper care, I meant proper medical supervision of the child, and a thorough knowledge of the child's family history, so that the best measures might be taken to ward off the hereditary evils, which, like the evil spirits of which we read, are in constant attendance upon the helpless children, waiting to pounce upon them just as soon as some weakness or injury shall give the opportunity. Syphilis and tuberculosis leave their impress upon the children unto the third and fourth generation, but these tendencies can be successfully combated, even in the first, and it is our duty to study constantly all the conditions and all the signs of dyscrasiæ, of development, and of diathesis, that we may know what we have to combat, and may have our weapons ready and burnished. The demons of disease and ill health have fastened themselves pretty strongly upon unfortunate humanity, and the best-directed efforts are feeble enough against them. But that does not excuse us from the eternal vigilance which is the price of nearly everything worth having in this life. I think that it is Professor Peirce who has pointed out that the teeth can be altered and developed by proper exercise, care and food. He alludes to the fact that the larvæ of the honey-bee are developed into a queen bee or into working bees, according to the nature of the food they receive. As to practical suggestions, I have purposely avoided discussions of the treatment of particular diseases as I have gone along, fearing to make my paper too long, and because the treatment of special conditions is unimportant compared to the hygiene of child life. Give a young child twelve hours sleep, plenty of sunlight, good air, and proper food containing plenty of phosphates, and he will develop that reserve of nerve-force which is needed to carry him through the emergencies of development.

Of the third period of our division a very great deal can be said. The hardships and vicissitudes experienced in getting the wisdom teeth and the general worthlessness of the product have engaged abler pens than mine, and I take it for granted that you are all more familiar with this part of our subject than with what has gone before, inasmuch as people suffering with their wisdom teeth are more apt to consult the dentist than the children and youth of whom we have just spoken; and also because of the more obvious and immediate connection of various troubles and the eruption or non-eruption of the wisdom teeth. However, there are certain physiological facts connected with this process which are not so generally known and which may profitably take up our time for a few moments longer.

There are many cases of eczema and other skin disease, for example, which attack the young, and which are often incurable until after the eruption of the third molars. You are all familiar with the acne of young people which, commonly speaking, gets better and worse again during a period of years, but is finally cured after the completion of the second dentition. The connection of eczema with the eruption of the teeth is, like tooth-rash in the first dentition, more obvious, and it is probably more tractable to treatment when the irritation of teething is over than the acne.

The nervous diseases connected with the cutting of the wisdom teeth are even more pronounced than those that come earlier in life, and the reflex neuroses are at this time more marked. You are all probably familiar with some of the numerous recorded cases of complete blindness of one or both eyes, of deafness, of spasms, migraine, chorea, epilepsy, and insanity due to impacted wisdom teeth. Some of the functional disorders connected with the wisdom teeth are perhaps not so familiar. Dr. Anstie mentions a case of severe uterine neuralgia clearly found to be due to a carious tooth, which was not itself at all sensitive. A case of alarming uterine colic in child-bed was, after ineffectual treatment, found to be due to an unerupted wisdom tooth, and was cured by the free use of the lancet over this tooth.

A curious case of vesical irritability is recorded in which the patient experienced pain in the teeth upon assuming the erect posture, especially when the feet touched the floor on arising in the morning.

Dr. Garretson gives a case of severe pain in a lower bicuspid tooth which was not relieved by extraction of the tooth, but was finally cured by curing an erosion on the inner surface of the fundus of the uterus. Hundreds of similar instances of reflex symptoms have been recorded. Dr. Mulveany says that he was frequently consulted by anxious young husbands because their youthful partners did not conceive. He always assured them that when their wives got through their teething they would have children, but that they were not likely to become mothers until quite over the infirmities of childhood.

I think that enough has been said to show that the eruption of the permanent teeth is by no means an unimportant process so far as it affects the health. Dr. Kingsley says that a perfect dental development is the result of well-balanced physical and nervous systems, without hereditary taint. "Abnormalities of development are due to disturbance of the trigeminal nerve during the period of the formation of the crowns of the permanent teeth. Neither lunacy nor insanity can have any direct bearing upon the development of the teeth, but would be most potent for evil if transmitted." The doctor continues:

"I do not hesitate to place it upon record that the next generation will see more of abnormality in dental development and an increase of nervous and cerebral diseases, and that the two are correlated and spring from the same cause, are not, strictly speaking, symptomatic of each other, but are associated and frequently have a common origin."

As dental irregularity is often accompanied by intellectual precocity, it may be said to be a sign of nervous excess in such persons.

As we glance backward over our subject we observe that the teeth of the permanent set which give the most trouble as they come through are those which do not replace milk teeth, *i. e.*, the permanent molar teeth. Their eruption may then be said to resemble the eruption of the milk teeth, and so it does. Convulsions, diarrhœas catarrhal diseases, etc., are more apt to show themselves with those than with any other of the permanent teeth. Of these teeth, also, it may be fairly said that they resemble milk teeth in being of softer consistency and more susceptible to caries than the rest of the permanent set. This is surely true of the first and third molars. Many theories have been advanced to account for the perishable character of the first and third permanent molars. Perhaps the first are poor because they are erupted so early, some claiming that they should not strictly be called permanent teeth, that they are a sort of link between the two sets, and that they should be extracted in time to make room for the third molars, which, it is claimed, would be good teeth if they had a chance to come through the gums promptly and easily, and could be kept clear and free from crowding.

This is a pretty theory, but scarcely a tenable one. Our jaws are small, our teeth poor and crowded, chiefly because we eat too highly-prepared foods, and do not exercise ourselves enough in chewing.

Our growth should be ever peaceful and regular. The constant talk about over-strain, neurasthenia, etc., only means that some one has sinned, either this man or his parents, and I judge it to be generally the latter, that he (or she) is born with "nerves." If we lay the foundation well for our children and teach them to avoid our mistakes in matters of hygiene we shall confer upon them benefits that wealth or learning cannot give. What I am constantly protesting against is the one-sided view of education that Americans entertain. We must educate—*i. e.*, draw out—the body and the mind, we must develop the chest, the arms, the backs, the jaws and teeth of our children, if we wish book-learning to be of any value to them or wish them to get any permanent good out of life or be of use in their day and generation.

Physical laws are God's laws, and if they are disregarded the punishment is swift and sure.

However, I am not one who takes a gloomy view of the physical future of the American people. I understood Professor Truman to say in your meeting last year that the teeth of our people are constantly growing better. I believe this to be true, and I believe that to our excellent and conscientious dentists much of the credit for this improvement is due.

When the laws of health are better understood, when the signs of a feeble constitution are more quickly read, when our people learn that building up and developing a normal body is really a simple thing, the Americans will become the finest race on the face of the earth.—*International Dental Journal.*

